# Factors influencing adherence to the food by prescription program among adult HIV positive patients in Addis Ababa, Ethiopia: a facility-based, cross-sectional study

**DOI:** 10.1186/2049-9957-3-20

**Published:** 2014-06-30

**Authors:** Mesrach Ayalew Kebede, Jemal Haidar

**Affiliations:** 1 St. Paul’s Hospital Millennium Medical College, Addis Ababa, Ethiopia; 2 School of Public Health, Addis Ababa University, P.O. Box 27285/1000, Addis Ababa, Ethiopia

**Keywords:** Factors, Adherence, Food by prescription program, HIV positive, Addis Ababa, Ethiopia

## Abstract

**Background:**

One way of addressing malnutrition among HIV/AIDS patients is through the Food by Prescription program (FBP) and many studies have explained the treatment outcomes after therapeutic food supplementation, though available evidences on adherence levels and factors associated with these sorts of programs are limited. The findings of this study would therefore contribute to the existing knowledge on adherence to Ready-to-Use Therapeutic/Supplementary Food (RUF) in Ethiopia.

**Methods:**

A facility-based, cross-sectional study supplemented with qualitative methods was conducted among 630 adult HIV + patients. Their level of adherence to RUF was measured using the Morisky 8-item Medication Adherence Scale (MMAS-8). The total score on the MMAS-8 ranges from 0 to 8, with scores of <6, 6 to <8, and 8 reflecting low, medium, and high adherence, respectively. Patients who had a low or a moderate rate of adherence were considered non-adherent.

**Results:**

The level of adherence was found to be 36.3% with a 95.0% response rate. With the exception of the educational status, other socio-demographic variables had no significant effect on adherence. Those who knew the benefits of the FBP program were 1.78 times more likely to adhere to the therapy than the referent groups. On the other hand, patients who were not informed on the duration of the treatment, those prescribed with more than 2 sachets/day and had been taking RUF for more than 4 month were less likely to adhere. The main reasons for non-adherence were not liking the way the food tasted and missing follow-up appointments. Stigma and sharing and selling food were the other reasons, as deduced from the focus group discussion (FGD) findings.

**Conclusion:**

The observed level of adherence to the FBP program among respondents enrolled in the intervention program was low. The major factors identified with a low adherence were a low level of education, poor knowledge on the benefits of RUF, the longer duration of the program, consuming more than two prescribed sachets of RUF per day, and not being informed about the duration of the treatment. Therefore, counseling patients on the program’s benefits, including the treatment plans, would likely contribute to improved adherence.

## Multilingual abstracts

Please see Additional file 1 for translations of the abstract into the six official working languages of the United Nations.

## Background

The human immunodeficiency virus/acquired immune deficiency syndrome (HIV/AIDS) is a global pandemic. By the end of 2011, 1.7 million patients, among the 34 million people living with HIV (PLHIV), died from the disease. Sub-Saharan Africa remains the most severely affected area, accounting for 69.0% of all cases worldwide [1]. According to the 2011 Ethiopian Demographic and Health Survey (EDHS), 1.5 percent of adults aged 15–49 years were reported to be infected with HIV [2]. Malnutrition and HIV infection have a direct relationship as malnutrition increases susceptibility to the HIV infection [3]. Together, HIV/AIDS and malnutrition may put PLHIV at an even greater risk of morbidity and mortality [4]. Other than this, PLHIV are vulnerable to having a poor nutritional status because their body’s nutrient requirements increase [5]. This refers to an increase in the recommended intake levels for healthy non-HIV-infected individuals of the same age, sex, and physical activity level [6].

Nutritional interventions have been successful in the management of HIV and AIDS, and many patients enrolled into such programs have markedly improved both their body weight and general health [7]. The Food by Prescription program is one of the strategies that addresses undernutrition among PLHIV and their vulnerable family members through nutritional assessment, counseling, and support (NACS) [8,9]. Different countries are at different stages of NACS programming, with Ethiopia and Tanzania beginning these programs in 2010 [10]. The FMoH of Ethiopia has launched a comprehensive National Nutrition Program, which includes nutrition and HIV/AIDS as part of its complete service delivery, and also emphasizes the importance of linking nutrition and HIV/AIDS programs with other livelihood programs [11].

Adequate nutrition is necessary to maintain the immune system, manage opportunistic infections, optimize response to medical treatment, sustain healthy levels of physical activity, and support optimal quality of life for PLHIV [12]. Cognizant of this fact, Ethiopia has integrated HIV and nutrition interventions based on the patient’s nutritional status. Ready-to-Use Foods (RUF) come in two forms: Ready-to-Use Therapeutic Foods (RUTF) and Ready-to-Use Supplementary Foods (RUSF). Both are nutrient-dense foods with a multitude of ingredients which require no preparation and are usually packaged in individual doses [9,13].

Despite the benefits of RUF, high default and loss of follow-up rates after enrollment in the program has been documented in some studies [14,15]. Other challenges of the program are that it takes a longer time to complete because of food sharing [8,16,17], stigma, household food insecurity, disliking of the RUF’s taste, as well as other factors related to the program design. These are all associated with the level of adherence among beneficiaries [18-22].

The Food by Prescription program is scaling up throughout Ethiopia, however, information related to its adherence among adults is scarce. This study was done to assess the situation and provide evidence-based information for both program managers and health providers about the existing challenges influencing adherence to RUF and the way forward.

## Methods

A facility-based, cross-sectional study with an analytical component supplemented by a qualitative study was conducted in 34 facilities in Addis Ababa, the capital city of Ethiopia, from February to June 2013. Addis Ababa had an estimated population of 3.43 million in 2013 [23]. The city is fully urban, with no rural dwellers within the city’s administrative boundaries and an estimated area of 526.99 square kilometers. From the 55 health facilities providing nutritional services for PLHIV in Addis Ababa, only 10 governmental hospitals and 25 health centers had started the Food by Prescription program and had a large client flow. These facilities were all included in the study except for one health center which discontinued the program during the data collection stage.

Ethical clearance was obtained from the research ethics committee of the School of Public Health at the College of Health Science, Addis Ababa University. The regional health office and the ethical review committees of the participating hospitals also cleared the study at their institutional levels. Individuals were enrolled in the study after they gave their informed and written consents. Consents ensured that participants understood the following: possible risks and benefits, that the participation was voluntary, assurance of confidentiality, the purpose of the research, how he/she was chosen to participate, data collection procedures, and whom to contact when questions and concerns arise (with the relevant contact details).

Participants included in the study were HIV positive (HIV+) adults already enrolled in a nutrition program (NP) for eight weeks who agreed to participate. Pregnant and lactating mothers enrolled in a NP and patients who could not stand straight when their height was measured were excluded.

### Quantitative study

Adding an estimated 5% for the non-response rate, a total of 630 participants made up the sample size, estimated based on an absolute precision of 4.0% with a 95% confidence level, and 50.0% prevalence. The numbers of patients proportional to the client flow were 254 from hospitals and 376 were from health centers. All the study subjects were interviewed for 30 minutes when coming to their antiretroviral therapy (ART) clinic to collect their RUF.

Pre-tested interviewer-administered structured questionnaires were used for the data collection. The important variables included in the questionnaire were the level of adherence (measured by the MMAS-8), socio-demographic variables, household food insecurity, information on therapeutic feeding, duration of therapy, and nutritional status of the patients registered at the time of entry into nutritional programs. Thirty-four data collectors (health officers and nurses) with relevant experience were recruited and trained for two days on the method of the data collection. The training addressed issues such as the content of the questionnaire, basic interviewing skills, and filling out of the questionnaire.

Data were edited manually initially, and then entered and organized using Epi Info version 3.5.1 and exported to SPSS version 20 for descriptive and inferential analyses. The results are presented in percentages and graphs where appropriate. Binary logistic regression was employed to examine the associations between the outcome variables (adherence) with the various independent factors (socio-demographic variables, household food insecurity, information on therapeutic feeding, duration of therapy, and nutritional status of patients), and the results are presented using crude odds ratios (CORs) and confidence intervals (95% CI). To ascertain the association between the dependent variables and the explanatory variables, simultaneously controlling for the aforementioned explanatory variables, stepwise logistic regression was applied and adjusted odds ratios (AORs) and confidence intervals (95% CI) were constituted. In all analyses, *P*< 0.05 was considered to be statistically significant.

### Qualitative study

Four focus group discussions (FGDs), two with six participants each from hospitals and two with six participants each from health centers (12 females and 12 males), who were willing and able to share their ideas, were conducted (a similar inclusion criteria was applied for the quantitative study). Discussion guides were used for the FGDs to gather detailed information on the issues until the information was saturated. Efforts were made to make the participants as homogeneous as possible. The principal investigator was assisted by a note taker who moderated the discussion and tape recorded all sessions. All notes and audio tape recordings of the interviews were fully transcribed, then analyzed by coding and identifying themes using the Open Code program version 3.6.2.0.

### Operational definitions/measurements

• Nutritional classification: The patients’ nutritional statuses were classified after their body mass index (BMI), where weight (kg) is divided by height in (meters) squared, was calculated. The nutritional status classified using BMI (kg/m^2^) is as follows: Severe Acute Malnutrition (SAM) (<16), Moderate Acute Malnutrition (MAM) (≥16 to <17), mild malnutrition (≥17 to <18.5), normal (≥18.5 to <25), overweight (≥25 to <30) and obese (≥30) [24].

• Ready-to-Use Food (RUF) includes both RUTF and RUSF, which are nutrient dense foods packed in sachets. RUTF as compared with RUSF provide larger quantities of energy and micronutrients needed for treating patients classified as SAM [25].

• Intake of RUF/day: To meet their additional daily energy requirements, patients received four sachets of RUTF (2000 kcal; Plumpy’ nut) per day if they were classified as SAM and two sachets of RUSF if they were classified as MAM, and/or exhibiting significant weight loss or demonstrating signs or symptoms of a disease. Patients had a monthly nutritional follow-up before renewal of their RUF prescription [11].

• Duration of therapy: Severely malnourished adults stay in nutritional programs for a minimum of four months and a maximum of six months, being supplemented with RUTF for the first two to three months and continuing with RUSF for the next two to three months. Those admitted to the nutritional program with a classification of MAM, exhibiting signs or symptoms of a disease, or who lost 5% of their body weight remain in the program two to three months taking RUSF [11].

• The mechanism used to measure adherence to RUF was based on patients’ self-reported answers in response to the specific questions using the new Morisky 8-item Measurement Assessment Scale (MMAS-8), which was developed from a previously validated four-item scale and supplemented with additional items to better capture information regarding the barriers surrounding adherence behavior. The new scale has been determined to have a higher reliability compared to the four-item scale (α = 0.83 versus 0. 61) [26], and is a self-reported questionnaire with eight questions (items). The total score on the MMAS-8 can range from 0 to 8, with scores <6 marking low level of adherence, 6 to <8 a moderate rate of adherence, and 8 reflecting a high adherence. Patients with a low or moderate rate of adherence were categorized as non-adherent [27,28].

• The Household Food Insecurity Access Scale (HFIAS) was used to assess the food insecurity status. The questions represented universal domains of a household’s food insecurity (access) experience and are used to measure households and populations along a continuum of severity. The scale ranges from food secure to severely food insecure. The respondent was first asked an occurrence question, i.e. whether the condition in the question happened at all in the past four weeks (yes or no). If the respondent answered “yes” to an occurrence question, a frequency-of-occurrence question is asked to determine whether the condition happened rarely (once or twice), sometimes (three to ten times), or often (more than ten times) in the past four weeks. In this study, only the occurrence questions were asked and only those households with food security and food insecurity of any degree were identified by considering only the “no” answer of the occurrence question. This means that participants who answered “no” for the nine occurrence questions were classified as food secure since the condition of food insecurity never occurred [29].

• Knowledge of the benefits of the Food by Prescription program referred to both RUTF and RUSF, and was calculated by considering the mean answers of the participants about their benefits, which included bringing strength, helping patients resume work, gaining weight, and decreasing feelings of hunger. Respondents who scored below the mean score were considered to have a poor knowledge, while those with above the mean score were considered to have a good knowledge of the benefits of the Food by Prescription program.

## Results

Of the 630 recruited subjects, only 600 HIV + patients participated in the quantitative study (a 95% response rate). Of these, 218 (36.3%) adhered strictly to the prescribed doses of food. Table 1 shows the socio-demographic characteristics of the respondents. Their ages ranged from 18 to 76 years, with a mean (SD) of 35.2 ± 10.1 years. Over half of the respondents (57.0%) were female and less than one third (30.7%) were in a marital union. The vast majority (90.0%) had a family size of ≤5 household members and about half (49.0%) had an educational level of secondary and above. The majority (80.1%) were Orthodox Christian, 234 (39.0%) had a private business, and 331(55.2%) were earning a monthly income of less than or equal to 500 Ethiopian Birr.

**Table 1:** Socio-demographic characteristics of participants enrolled in the Food by Prescription program in Addis Ababa, Ethiopia, 2013

***Variable***	***Frequency***	***Percent***
**Age (in years)**		
18–29	182	30.3
30–39	255	42.5
40–49	109	18.2
>50	54	9.0
Mean 35.2 ± 10.1		
**Sex**		
Female	342	57.0
Male	258	43.0
**Marital status**		
Single	206	34.3
Marital union	184	30.7
Non-marital union	210	35.0
**Family size**		
≤5 (nuclear family)	540	90.0
>5 (extended family)	60	10.0
**Education**		
No school	101	16.8
Primary	205	34.2
Secondary	235	39.2
Tertiary	59	9.8
**Religion**		
Christian	555	92.5
Muslim	45	7.5
**Occupation**		
Private business	234	39.0
Employed	366	61.0
Income (in Birr*)		
≤500	331	55.2
501–999	156	26.0
>1000	113	18.8

*20 Birr = 1 USD.

Table 2 shows the baseline information of participants enrolled in a nutrional program. The majority (72.6%) perceived that RUF was only given to HIV + adults and children, less than half (44.3%) thought that the food was given to PLHIV who experienced weight loss, and about a third (31.0%) said that the food is given to all malnourished adults and children. A hundred and five (17.5%) didn’t attend their regular follow-ups and did not collect the food because they were not told to do so. About three quarters (73.2%) were informed about the duration of the treatment. Most of the participants (71.7%) were prescribed with <2 sachets/day and about two thirds (63.3%) had stayed in the program for <3 months. About one third (34.3%) reported that their appetite was not tested when they were first prescribed the food and over half (54.6%) were food insecure. The proportion of participants classified as SAM, MAM, and losing a significant amount of weight and having an aggravating disease was 183 (30.5%), 378 (63.0%), and 39 (6.5%), respectively.

**Table 2:** Baseline information of the participants enrolled in the Food by Prescription program in Addis Ababa, Ethiopia, 2013

***Variable***	***Frequency***	***Percent***
RUF beneficiaries* (Patients’ perceptions)		
Given only to HIV + adults and children	436	72.6
All malnourished children and adults	186	31.0
HIV + adults with weight loss	266	44.3
HIV + adults with a sign and symptomatic disease	83	13.8
I don’t know	33	5.5
**Had regular follow-ups**		
Yes	495	82.5
No	105	17.5
Reason for not coming*		
Not told	44	41.9
Not necessary	15	15.2
Far from home	17	16.2
Others^a^	29	30.5
**Informed on treatment duration**		
Yes	439	73.2
No	161	26.8
**Prescribed RUF per day**		
≤2 sachets	430	71.7
>2 sachets	170	28.3
**Duration of stay in the program (in months)**		
<3	380	63.3
3–4	121	20.2
≥4	99	16.5
**Appetite test done before enrolment**		
Yes	394	65.7
No	206	34.3
**Food security status**		
Food insecure	328	54.6
Food secure^b^	272	45.4
**Nutritional status**		
SAM	183	30.5
MAM	378	63.0
Weight loss and symptomatic diseases	39	6.5

*values exceeded 100 percent because of multiple responses; a = includes social reasons like death of family member, being busy, illnesses, transportation cost and side effects; b = answered no for the 9 occurrence question; RUF (Ready-to-Use Foods); SAM (Sever Acute Malnutrition); MAM (Moderate Acute Malnutrition).

Table 3 shows factors associated with the level of adherence. With the exception of the educational status, other socio-demographic variables had no significant affect on adherence. Those who had primary, secondary, and tertiary education were more than two times likely to adhere to the prescribed food than the referent groups. Respondents who knew the benefits of the prescribed food were 1.78 times (AOR 1.78; 95%CI 1.22 to 2.60, p = 0.002) more likely to adhere than those who didn’t know the benefits. Patients who were not informed on the duration of the nutritional therapy and prescribed with more than two sachets/day were 61% (AOR 0.39; 95% CI 0.24 to 0.63) and 47% (AOR 0.53; 95% CI 0.33 to 0.85), respectively, less likely to adhere and consume the prescribed food than their counter participants. Similarly, participants who had been consuming the food for more than four months were 62% less likely to adhere. On the other hand, patients who had an appetite test before admission to the program and households that were food insecure were significantly associated with adherence only in the bivariate analysis.

**Table 3:** Factors associated with the level of adherence in participants enrolled in the Food by Prescription program in Addis Ababa, Ethiopia, 2013

***Variable***	***Good adherence N (%)***	***Low adherence N (%)***	***COR (95% CI)***	***AOR (95% CI)***
**Knowledge of benefits**				
Poor	77 (12.8)	201 (33.5)	1	1
Good	141 (23.5)	181 (30.2)	2.03 (1.44–2.86)*	1.78 (1.22–2.60)*
Knew treatment duration**				
Yes	185 (30.8)	254 (42.3)	1	1
No	33 (5.5)	128 (21.3)	0.35 (0.23–0.54)*	0.39 (0.24–0.63)*
**Prescribed RUF**				
**≤**2 sachets/day	173 (28.8)	257 (42.8)	1	1
>2 sachets/day	45 (7.5)	125 (20.8)	0.53 (0.36–0.79)*	0.53 (0.33–0.85)*
**Appetite test done**				
No	63 (10.5)	143 (23.8)	1	1
Yes	155 (25.8)	239 (39.8)	1.47 (1.02–2.10)*	1.28 (0.85–1.94)
**Duration of intake**				
<3 months	155 (25.8)	225(37.5)	1	1
3–4 months	40 (6.6)	81(13.5)	0.71 (0.46–1.10)	0.65 (0.40–1.05)
≥4 months	23 (3.8)	76(12.6)	0.43 (0.26–0.73)*	0.38 (0.22–0.68)*
**Food security**				
Food insecure	108 (18.0)	220 (36.6)	0.72 (0.51–1.01)*	0.80 (0.54–1.17)
Food secure	110 (18.3)	162 (27.0)	1	1
**Knew current weight**				
Yes	213 (35.5)	357 (59.5)	1	1
No	5 (0.8)	25 (4.2)	0.33 (0.12–0.88)*	0.49 (0.16–1.46)
**Education**				
No school	20 (3.3)	81 (13.5)	1	1
Primary	72 (12.0)	133 (21.2)	2.19 (1.24–3.86)*	2.69 (1.42–5.09)*
Secondary	98 (16.3)	137 (22.8)	2.89 (1.66–5.04)*	2.80 (1.48–5.30)*
Tertiary	28 (4.6)	31 (5.2)	3.65 (1.80–7.42)*	3.41 (1.53–7.58)*
**Patients enrolled in**				
Hospitals	101 (16.8)	143 (23.8)	1	1
Health centers	117 (19.5)	239 (39.8)	0.69 (0.49–0.97)*	0.77 (0.52–1.13)
**Nutritional status**				
SAM	55 (9.2)	128 (21.3)	1	1
MAM	148 (24.7)	230 (38.3)	1.49 (1.02–2.18)*	0.90 (0.56–1.44)
Weight loss and symptomatic disease	15 (2.5)	24 (4.0)	1.45 (0.70–2.98)	0.66 (0.29–1.52)

*p < 0.05, **treatment duration for RUF (Ready-to-Use Foods); RUF (Ready-to-Use Foods); SAM (Sever Acute Malnutrition), MAM (Moderate Acute Malnutrition), COR (crude odd ratio), AOR (adjusted odd ratio).

**Figure 1:**
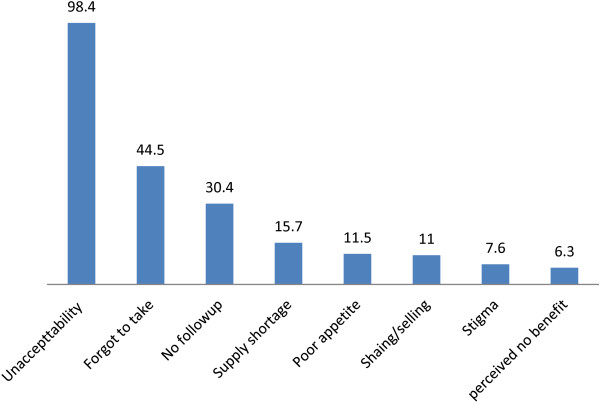
Types of reasons mentioned to discontinue the food, Addis Ababa, Ethiopia, 2013.

**Figure 2:**
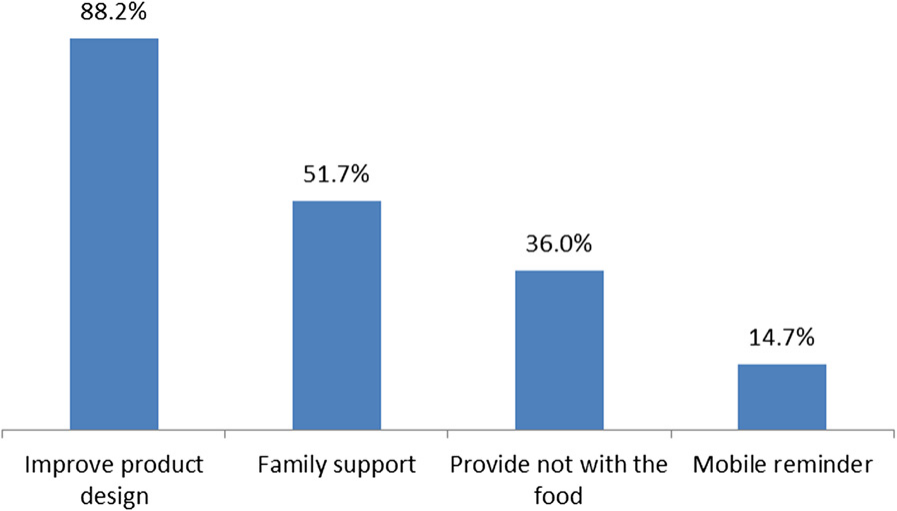
Types of suggestions forwarded to improve level of adherence to the food by prescription program, Addis Ababa, Ethiopia, 2013.

**Figure 3:**
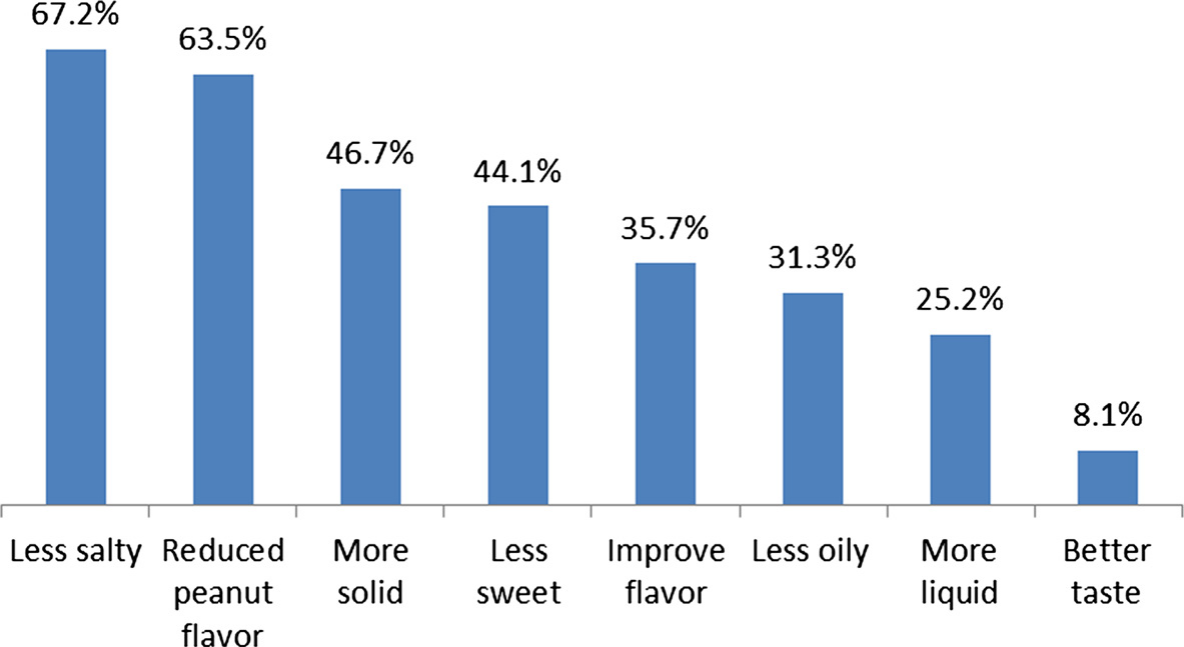
Types of suggestion forwarded by participants to improve the product design, Addis Ababa, Ethiopia, 2013.

The level of adherence was significantly associated with the type of facilities that patients were enrolled in and those who knew their current weight status although the association was absent in the multivariate analysis. Furthermore, there was no association of adherence with other factors such as whether the patients had symptomatic diseases, exhibited significant weight loss, their ART status, or whether they received organizational support. Other factors such as duration of illness, disclosure of HIV status, whether patients were on ART or not, presence of a reported disease, and those who lost weight in the past two months had no significant association with adherence to the prescribed food.Figure 1 shows the various reasons why respondents discontinued or minimized taking the prescribed foods. The leading reason for non-adherence reported by the majority (98.4%) was disliking the product followed by forgetfulness (44.5%), not attending follow-ups (30.4%), supply problem in the facility (15.7%), poor appetite (11.5%), sharing and selling of the product (11.0%), and stigma (7.6%).Figure 2 shows the suggestions forwarded to enhance the level of adherence to the prescribed food. From 390 participants suggesting means to improve adherence, 344 (88.2%) suggested a change in the product design and 259 (66.4%) suggested getting a reminder by other means, 134 (51.7%) mentioned family support, 93 (36.0%) said a written note with the prescribed food would help, and 38 (14.7%) thought a mobile reminder would enhance adherence.Figure 3 shows some of the suggestions made by the participants to improve the product design. From 344 respondents suggesting change in the product design, the majority (72.0%) wanted to change the taste of the product, with 189 (67.2%) preferring it to be less salty, 126 (32.3) and 214 (54.8%) suggested a change in the smell and the consistency of the product, respectively, from which 80 (63.5%) wanted to reduce the peanut smell and 100 (46.7%) requested a more solid consistency of the product, respectively.

### Qualitative results

According to the qualitative findings, the major themes identified were perception and adherence. Although patients had positively perceived the benefits of RUF, they had low levels of adherence due to perceived barriers such as stigma, undesirable taste/unacceptability of the product, sharing and selling of the product, and the program design. All these affected the patients’ adherence along with the low perceived susceptibility to malnutrition (data not shown).

### Perception about RUF

Most of the FGD participants perceived the benefit of the food commodity positively and all mentioned that the food helps them to gain weight and strength, and allows them to resume their work. Other than that, the food was described as being made out of many components which help patients regain their appetite, increase their CD4 count, rebuilt their bodies, and ultimately improve their quality of life. In addition, some participants said that the food commodity was like medicine given to a person when he/she is malnourished.

### Adherence to RUF

Four out of the 24 FGD participants thought a person who did not consume the prescribed food as advised by the health workers will not gain weight as expected and delay recovery. Although the majority of the participants agreed that taking the food as prescribed was not difficult, some mentioned that they defaulted because of perceived barriers which included stigma, fasting, sharing and selling of RUF, disliking the taste, disease conditions, and program design.

### Stigma related with RUF

Some of the participants had difficulty adhering to RUF because they felt stigmatized as the food is strongly associated with HIV, and people easily identify their sero positivity. One of the female participants said: *“Yes, sometimes it might be difficult to pick up the food and consume it. For example, I don’t take the plumpy’ nut when there are people in my house because I feel like they might identify me as being HIV positive. I don’t even throw out the empty sachets in my house so that no one can see them and always take the sachets to the health center and dispose of them there.”*

### Sharing and selling of RUF

Eight out of the 24 participants said that they share the prescribed food with their children because their children like it and they cannot avoid such practices because of their culture. Poor adherence was also linked to sharing with other HIV + friends with whom they share similar health conditions such as weakness and severe weight loss. One of the participants said: “*I used to share RUF with my friend; she is also positive and had the same health conditions as me, such as weakness and diarrhea. She didn’t attend follow-ups; she was just taking the holy water. So I used to share the food with her for weeks.”* Selling was also mentioned as another factor affecting bad adherence among some beneficiaries though the majority of the participants said they did not sell their share.

### Disliking the product (RUF)

Almost all the participants said that the taste and the consistency of the product was the reason they minimized or discontinued the RUF. A male respondent from the third FGD said: *“I would prefer the food if it was solid in consistency as it disgusts me to take it when it is a liquid.”*

### Program design

Almost all the participants said that they were counseled about the benefits and how to take the RUF before being enrolled in the program. Facilities providing RUF had their own systems of handling some of the issues related with RUF intake and preferred to prescribe the amount of RUF only for 15 days, or schedule separate appointments from the ART so that the RUF wouldn’t be misused. Nonetheless, the provider tended to forget and only prescribed the one-month drug refill.

### Conditions enhancing RUF intake

Some patients believed that having family support and counseling helped them to take the prescribed amount even when they were experiencing side effects or forgetting to take the RUF. One of the female participants said: “*Whenever I forget, my son always reminds me to take it.”*

## Discussion

The present study identified a number of factors that affect patients’ levels of adherence to prescribed doses of RUF. The major factors associated with adherence were the educational status, knowledge of benefits, duration of the nutritional program, prescribed number of sachets per/day, and availing information on the duration of the therapy.

As the level of education increased, the adherence to the prescribed RUF also increased, with a tertiary education resulting in adherence being three times more likely. Likewise, patients who knew the benefits of RUF were 1.78 times more likely to adhere than their referent groups which was also reflected during the FGDs. These results conform with a Kenyan study [21].

Patients who were not informed on the duration of therapy were 61% less likely to adhere than their counterparts and the difference noted was significant. This observation was also concordant in the qualitative findings where patients considered the treatment to be given for a month and simply come for the ART follow-up after three months. The prescribed amount of food for the day was also significantly associated with the level of adherence. Patients who prescribed the food for more than four months were 62% less likely to adhere than their counterparts and the difference was statistically significant. This finding was, however, different from the Kenyan study where the ‘first three or four days were the most critical ones and then it becomes easier’ to comply with the prescription [21]. Patients prescribed more than two sachets/day were 47% less likely to adhere and this is concordant with the Kenyan study where non-compliance was observed in patients who were prescribed four sachets/day [17]. These two findings show that patients are probably fed up taking the food so frequently in addition to the long duration of the Food by Prescription program.

The leading reason for non-adherence in both the quantitative and the qualitative analyses was disliking the product, and this conforms with the Kenya study where it was reported that the product was perceived to induce nausea and vomiting because of the taste [21]. Another study done in Bangladesh also supports the present findings where taste was mentioned to induce similar symptoms [20]. The taste, the smell, and the consistency of the product were not desirable for patients to consume and affected their level of adherence. Other than this, forgetting (44.5%), not attending follow-ups (30.4%), a supply problem in the facility (15.7%), poor appetite (11.5%), sharing and selling (11.0%), and stigma (7.6%) were some of other reasons affecting the adherence level in this study.

In a recent study conducted in Sub-Saharan Africa, among the HIV infected malnourished adults enrolled in the Food by Prescription program, the default rate was 22.6% [14]; this finding is lower than the present study findings (30.4%). In the present study, patients who defaulted reduced their daily intake to ensure that the amount received would last them until the next scheduled visit with the ART follow-up appointment, and this evidently could delay recovery. The attribute for the high default rate in the present study, other than what has been mentioned above, was the delay in distributing the food to the facilities as the food supply chain is managed by a government agency which is responsible for managing other medical supplies as well. This affects the adherence level, as well as the recovery [10].

In the Kenyan qualitative study, most patients did not know about the relationship between the HIV infection, their body weight, and ART therapy [21]. This finding was also observed during the FGDs where patients poorly perceived the relationship of the illnesses with their nutritional status, indicating that they overlooked the product and focused on the ART alone.

Poor appetite was mentioned as one of the reason for non-adherence. Those patients who did an appetite test when they were enrolled in the program were 1.28 times more likely to adhere than those who did not receive an appetite test, indicating the importance of an appetite test before starting the Food by Prescription program [13].

In the present study, the sharing and selling of RUF was another reason affecting adherence; a factor mentioned by most of the patients. This finding was consistent with some previous studies done elsewhere [8,17,21]. The acceptability and compliance assessment done among similar patients enrolled in the Food by Prescription program in Kenya showed that RUF was shared with HIV + non-malnourished partners [17]. Another investigation of adherence in the same country among adult AIDS patients found that patients shared the food (Plumpy’ nut) with family members. This was a common practice and the reason attributed was food insecurity at the household level. In addition to this, the patients’ children liked the food and asked for it every time the beneficiaries were consuming it, which ultimately led to poor adherence [21]. Similarly, in this study, food insecure households were 28% less likely to adhere. Other studies have also suggested that in households with severe food insecurity, the RUF is shared among family members [19]. Selling of the product for replacement with other food items in the household was also another reason mentioned in the FGDs that affected the level of adherence.

A socio-anthropological investigation related to the acceptability of the prescribed food (Plumpy’ nut) in Cambodia had indicated that a stigma related with the intake exists [18]. This study has also found stigma to be one of the reasons for non-adherence. This was discovered during the FGDs when the majority of the participants mentioned that they either missed or discontinued therapy in fear of being disclosed when collecting the food because the food is mainly associated with HIV + adults and children.

The study also identified factors that could enhance adherence among beneficiaries. The majority of the participants preferred the product to be less salty with a reduced peanut smell, and for it to be solid in its consistency. Similar suggestions were found in the Bangladesh study where improvement in the product design was also mentioned to improve adherence [20]. In this study, family support and written note reminders were also singled out as being able to help patients better adhere to the prescribed dose of RUF, highlighting the importance of family support.

## Conclusion

The observed level of adherence to the Food by Prescription program was low and the major contributory factors identified were: a low level of education, a poor perception of the benefits of RUF, a longer duration of the nutritional program, over two prescribed sachets of food per day, and being uninformed about the duration of the therapy. Other reasons for non-adherence were dislike of the product because of its consistency and forgetting to consume the food. Therefore, counseling patients on the benefits of RUF, including on the treatment plans, would contribute to an improvement in the Food by Prescription program, as well as to better adherence. Modifying the product design, family support, and written note reminders can help patients and ultimately improve adherence to the prescribed dose of RUF.

This study was the first of its kind, using a mix of quantitative and qualitative study designs including suggestions from the respondents as a means to enhance adherence. However, it was not easy to measure the temporal relationship since both exposure and outcome variables were collected simultaneously (self-reported measures of adherence tend to overestimate adherence and completely rely on the patients’ responses) and this was a limitation of the study. Other limitations included that the household food insecurity status was limited to the occurrence questions and that it would have been better to include the frequency of the occurrence to clearly determine the food insecurity status. The inclusion of healthcare providers would have uncovered some other factors related to patients’ adherence as healthcare providers are directly involved in the service.

## Competing interests

Both authors declare that they have no competing interest.

## Authors’ contributions

MKA conducted the study as her partial fulfillment for the MPH study and drafted the manuscript. JAH contributed in the formulation of the study question and supervised the entire study, including the data interpretation and revision of the manuscript for intellectual content. Both authors read and approved the submitted manuscript.

## Supplementary Material

Additional file 1 Multilingual abstracts in the six official working languages of the United Nations. Click here for file
