# Supplementary material for: Factors influencing adherence to the food by prescription program among adult HIV positive patients in Addis Ababa, Ethiopia: a facility-based, cross-sectional study
Source: Infect Dis Poverty. 2014 Jun 30;3:20. doi: 10.1186/2049-9957-3-20 (PMC4101708; doi:10.1186/2049-9957-3-20)
Supplement: Additional file 1 — Multilingual abstracts in the six official working languages of the United Nations. [file 2049-9957-3-20-S1.pdf]

Translation of the abstract into the six official working languages of the United Nations

العوامل المؤثرة على الالتزام ببرنامج التغذية بوصفة طبية بين المرضى البالغين المصابين بفيروس نقص المناعة البشرية HIV في أديس أبابا، أثيوبيا: دراسة عرضية بالمرافق الصحية

مسراش أياليو كبيدي ود. جمال حيدر

الخلاصة

الخلفية: قد كانت التدخلات الغذائية ناجحة في إدارة فيرس نقص المناعة البشرية HIV والإيدز AIDS، والعديد من المرضى المسجلين في البرامج الغذائية قد تحسن وزن أجسامهم وصحتهم العامة بشكل ملحوظ. طريقة واحدة لمعالجة سوء التغذية بين مرضى فيروس نقص المناعة البشرية والإيدز AIDS/HIV من خلال برنامج التغذية بوصفة طبية. وقد أوضحت العديد من الدراسات نتائج استخدام المكملات الغذائية العلاجية، ولكن توجد دراسات محدودة عن مستويات الالتزام والعوامل المرتبطة بهذه الأنواع من البرامج. ولذلك فإن نتائج هذه الدراسة تساهم في المعرفة الحقيقية عن الالتزام ببرنامج الغذاء التكميلي / العلاجي الجاهز للاستخدام (RUF) في أثيوبيا.

الهدف: تقييم العوامل المؤثرة على الالتزام ببرنامج التغذية بوصفة طبية بين المرضى البالغين المصابين بفيروس نقص المناعة البشرية HIV والمسجلين في برامج غذائية في المرافق الصحية بأديس أبابا.

طرق العمل: كانت الدراسة عبارة عن دراسة عرضية بأحد المرافق الصحية مستوفاة بطرق عمل نوعية، أجريت في المرافق الصحية بأديس أبابا بين فبراير ويونيه 2013. تم تسجيل ما مجموعه 360 مريض بالغ بفيروس HIV من 34 مرفق صحي وتم تسجيل مستوى الالتزام ببرنامج الغذاء التكميلي / العلاجي الجاهز للاستخدام (RUF) باستخدام مقياس موريسكي للالتزام بالعلاج (MMAS-8). تم إدخال البيانات في برنامج Epi Info version 3.5.1 وتحليلها باستخدام برنامج SPSS version 20. يتراوح مجموع الدرجات على مقياس موريسكي للالتزام بالعلاج (MMAS-8) من 0 إلى 8 ، بدرجات <6، 6 إلى <8، و 8 موضحاً الالتزام المنخفض، والمتوسط والعالي على التوالي. المرضى دوي المعدل المنخفض أو المتوسط من الالتزام اعتبروا غير ملتزمين. وقد تم تحليل العوامل ذات الصلة بمستوى الالتزام باستخدام الانحدار اللوجستي المتعدد ذو الحدين.

النتائج: شارك ستمائة مريض في الدراسة الكمية بمعدل استجابة 95.0% ، ووجد أن مستوى الالتزام يبلغ 36.3%. باستثناء الحالة التعليمية، ليس للمتغيرات الاجتماعية الديموجرافية تأثيراً هاماً على الالتزام. أولئك الذين عرفوا فوائد برنامج التغذية بوصفة طبية كانوا 1.78 مرة (AOR=1.78; 95 %CI=1.22 to 2.60) أكثر عرضة للالتزام بالعلاج من المجموعات المرجعية. على الجانب الآخر، المرضى الذين لم يتناولوا الغذاء التكميلي / العلاجي الجاهز للاستخدام (RUF) لأكثر من أربعة أشهر كانوا 61.0% (AOR=0.39; 95%CI=0.24 to 0.63), 47% (AOR=0.53; 95%CI=0.33 to 0.85), and 62.0% (AOR=0.38; 95%CI=0.22 to 0.68) على التوالي، أقل عرض للالتزام بالعلاج من نظرائهم المشاركين. كانت الأسباب الرئيسية لعدم الالتزام عدم استساغة طعم الغذاء وفقدان مواعيد للمتابعة، وفقاً للنتائج الكمية. والأسباب الأخرى تمثلت في اعتبار هذا وصمة عار وتقاسم وبيع الغذاء، كما يستنتج من نتائج مناقشة مجموعة التركيز (FGD).

الاستنتاج: كان المستوى الملاحظ من الالتزام ببرنامج التغذية بوصفة طبية بين المستجيبين المسجلين في برنامج التدخل منخفضاً. تمثلت العوامل الرئيسية المرتبطة بانخفاض الالتزام في انخفاض مستوى التعليم، والمعرفة

المتواضعة بفوائد الغذاء التكميلي / العلاجي الجاهز للاستخدام (RUF)، والمدة الطويلة للبرنامج، واستهلاك أكثر من كيسين مقررين من الغذاء التكميلي / العلاجي الجاهز للاستخدام (RUF) كل يوم، وعدم المعرفة بمدة العلاج. لذلك، تقديم المشورة للمرضى عن فوائد البرنامج، بما في ذلك خطط العلاج، سيسهم على الأرجح في تحسين الالتزام.

Translated from English version into Arabic by Saher Salama, through

TRANSLATORS
WITHOUT BORDERS

埃塞俄比亚亚斯亚贝巴成年 HIV 阳性患者对饮食治疗项目（Food by Prescription program）依从性的影响因素研究

Mesrach Ayalew Kebede and Dr. Jemal Haidar

摘要

引言：在 HIV/AIDS 患者的管理中，营养干预措施已取得成功，许多参加营养干预项目的患者在体重和健康状况方面均有显著改善。饮食治疗项目是解决 HIV/AIDS 患者营养不良问题的一种方法。很多研究已经对饮食治疗项目的效果进行了阐述，但关于饮食治疗项目参与者依从性及其影响因素的研究报道较少。因此开展 HIV/AIDS 患者依从性及其影响因素的研究将有助于饮食治疗项目在埃塞俄比亚的顺利实施。

目的：评估亚的斯亚贝巴卫生机构中 HIV 阳性患者对饮食治疗项目依从性的影响因素。

方法：2013 年 2 月-6 月，以医院为基础的横断面调查研究在亚的斯亚贝的卫生机构中开展，共有 630 名来自 34 家卫生机构中的成年 HIV 阳性患者参加了本次研究。使用 Morisky 8 项药物依赖性量表（MMAS-8）测定研究对象对饮食治疗项目的依从性水平。MMAS-8 量表总得分范围从 0 到 8，得分小于 6、6～8 之间和 8 分别表示依从性低、中和高。那些得分为低或中度依从性的研究对象被认定为非依从性人群。采用二元多因素 Logistic 回归模型对与依从性水平的相关因素进行分析。

结果：共有600名患者参加了定量研究，响应率为95.0%，依从性水平为36.3%。除了受教育水平，其他社会人口学变量对依从性水平无显著影响。与对照组相比那些知道饮食治疗项目好处的研究对象优势比为 1.78(AOR=1.78; 95 %CI=1.22-2.60)，参与治疗依从性较好。另一方面，与对照组相比，参与饮食治疗项目少于4个月的研究对象的优势比分别是 61.0% (AOR=0.39; 95%CI=0.24-0.63), 47% (AOR=0.53; 95%CI=0.33-0.85), 和 62.0% (AOR=0.38; 95%CI=0.22 -0.68), 参与治疗的依从性较差。定量研究结果显示，依从性差的主要原因为不喜欢食物的味道和错过了随访时间。焦点讨论小组研究结果显示，依从性差的主要原因为歧视、分享和出售食物。

结论：参与干预项目的受访者对食疗项目的依从性水平较低。主要原因有受教育水平低、对饮食治疗项目的好处认识不足、项目持续时间过长、每天消耗超过两份饮食治疗项目提供的食物、未被告知治疗持续时间。因此，让参与者知晓参与项目的好处，了解详细的治疗方案将有助于提高参与者的依从性。

Translated from English version into Chinese by TIAN Li-guang, through

CDC CHINESE CENTER FOR DISEASE CONTROL AND PREVENTION

NATIONAL INSTITUTE FOR PARASITIC DISEASES

Facteurs influençant l'adhésion au programme «Alimentation sur ordonnance» (Food by Prescription program) chez des patients adultes séropositifs pour le VIH à Addis Abeba, Ethiopie: étude transversale réalisée dans les centres de soins.

Mesrach Ayalew Kebede et Dr Jemal Haidar

Abrégé

Contexte: Les actions portant sur la nutrition ont été positives dans la gestion du VIH et du SIDA et de nombreux patients inscrits dans des programmes nutritionnels ont vu leur poids augmenter et leur état de santé général amélioré de façon significative. Une manière d'aborder la malnutrition des patients VIH/SIDA passe par le programme « Alimentation sur ordonnance ». De nombreuses études ont détaillé les résultats du traitement après une supplémentation en aliments thérapeutiques, mais peu d'études portent sur les degrés et les facteurs de l'adhésion associés à ces types de programmes. Les résultats de cette étude étendraient par conséquent les connaissances actuelles concernant l'adhésion à la prise d'aliments thérapeutiques/compléments alimentaires prêts à l'emploi (ATPE) en Ethiopie.

Objectif: Déterminer les facteurs influençant l'adhésion au programme « Alimentation sur ordonnance » chez des patients adultes séropositifs pour le VIH (VIH+) inscrits dans des programmes nutritionnels dans des centres de soins d'Addis Abeba.

Méthodes: Il s'agissait d'une étude transversale, réalisée dans les centres de soins, complétée par des méthodes qualitatives, conduite dans des établissements de santé d'Addis Abeba entre février et juin 2013. Au total, 630 patients adultes VIH+ provenant de 34 établissements ont été inscrits et leur degré d'adhésion aux ATPE était mesuré par le questionnaire d'adhésion au traitement médicamenteux en 8 points de Morisky (MMAS-8). Les données étaient enregistrées dans la version Epi Info 3.5.1 et analysées en utilisant la version SPSS 20. Le score total auMMAS-8 s'échelonnait de 0 à 8, avec des scores inférieurs à 6 ; compris entre 6 et 8 ; et égal à 8 reflétant respectivement un degré d'adhésion bas, moyen ou élevé. Les patients qui obtenaient un niveau d'adhésion bas ou moyen étaient considérés comme non-adhérents. Les facteurs associés au degré d'adhésion étaient analysés en utilisant des régressions logiques binaires multiples.

Résultats: Six cents patients ont participé à l'étude quantitative avec un taux de réponse de 95,0% et le niveau d'adhésion était de 36,3%. A l'exception du niveau d'éducation, les autres variables sociodémographiques n'avaient pas de retentissement significatif sur l'adhésion. Ceux qui connaissaient les bénéfices apportés par le programme « Alimentation sur ordonnance » étaient 1,78 fois (OR ajusté = 1,78 ; IC95% =1,22 à 2,60) plus susceptibles d'adhérer au traitement que les groupes de référence. D'autre part, les patients qui ne prenaient pas d'ATPE depuis plus de

quatre mois étaient respectivement 61% (OR ajusté = 0,39 ; IC = 0,24 à0,63), 47% (OR ajusté = 0,53 ; IC95% = 0,33 à 0,85) et 62%(OR ajusté = 0,38 ; IC95% = 0,22 à 0,68) moins susceptibles d'adhérer au traitement que les autres participants. Les principales raisons de non-adhésion étaient de ne pas aimer le goût des aliments et de manquer les rendez-vous de suivi, selon les analyses quantitatives. La stigmatisation, le partage de la nourriture et la vente de la nourriture constituaient les autres raisons découlant des constatations des discussions de groupes.

Conclusion: Le degré d'adhésion observé au programme « Alimentation sur ordonnance » des personnes interrogées et inscrites dans le programme d'action était bas. Les facteurs majeurs identifiés contribuant à une faible adhésion étaient le niveau d'éducation bas, le manque de connaissances sur les bénéfices des ATPE, une plus longue durée du programme, le fait de consommer plus de deux sachets d'ATPE par jour et le fait de ne pas être informé de la durée du traitement. Par conséquent, les conseils aux patients sur les bénéfices de ce programme, y compris sur les plans de traitement, seraient susceptibles de contribuer à améliorer l'adhésion.

Translated from English version into French by Laurence Bezombes, through

TRANSLATORS
WITHOUT BORDERS

Факторы, влияющие на приверженность программе «Food by Prescription» («Еда по рецепту») ВИЧ-инфицированных пациентов в г. Аддис-Абеба (Эфиопия). Одномоментное межгрупповое исследование, проводимое в пределах учреждений.

Мезрах Айялеу Кебед и д-р Джемал Хайдар

Краткое изложение

История вопроса: Диетологическое вмешательство является успешным в работе с ВИЧ и СПИДом, и многие пациенты, зачисленные в диетологические программы, значительно улучшают свой вес и общее состояние здоровья. Одним из способов работы с истощением среди пациентов с ВИЧ/СПИДом является программа «Еда по рецепту». Многие исследования объясняют результаты лечения после применения продуктов лечебно-профилактического назначения, но недостаточно исследований по уровням приверженности и другим факторам, связанных с такими программами. Таким образом, результаты этого исследования будут полезны для пополнения знаний о приверженности программам готовой к использованию лечебно-профилактической еды/пищевых добавок (RUF) в Эфиопии.

Цель: Оценить факторы, влияющие на приверженность программе «Еда по рецепту» среди ВИЧ-инфицированных (ВИЧ+) взрослых пациентов, зачисленных в диетологические программы в медицинских учреждениях в г. Аддис-Абеба.

Методы: Данное исследование является одномоментным межгрупповым исследованием с использованием качественных методов, проведенным в медицинских учреждениях г. Аддис-Абеба в период с февраля по июнь 2013 г.. 630 взрослых ВИЧ-инфицированных пациентов из 34 учреждений были зачислены в программы, и их уровень приверженности был оценен с помощью 8-пунктной шкалы приверженности лечению Мориски (MMAS-8). Данные были введены в Epi Info версии 3.5.1 и проанализированы с помощью SPSS версии 20. Значения по MMAS-8 отражаются по шкале от 0 до 8, где значения <6, от 6 до <8 и 8 представляют собой низкую, среднюю и высокую приверженность соответственно. Пациенты с низкой и средней приверженностью рассматривались как неприверженные программе. Факторы, связанные с

уровнем приверженности, анализировались с помощью бинарных множественных логистических регрессий.

Результаты: 600 пациентов приняли участие в количественном исследовании с долей ответивших 95,0%, и уровень приверженности оказался равен 36,3%. За исключением образования, социально-демографические переменные не оказали существенного влияния на приверженность. Те, кто знали о преимуществах программы «Еда по рецепту», были в 1,78 раза (AOR=1,78; 95%CI=1,22-2,60) более склонны придерживаться лечения, чем референтные группы. С другой стороны, пациенты, участвующие в программах RUF не более четырех месяцев были на 61,0% (AOR=0,39; 95%CI=0,24-0,63), 47% (AOR=0,53; 95%CI=0,33-0,85) и 62,0% (AOR=0,38; 95%CI=0,22-0,68) соответственно, менее склонны придерживаться лечения, чем участники, участвующие в программах на протяжении более длительного времени. Согласно результатам количественных исследований основными причинами неприверженности является то, что пациентам не нравится вкус еды, а также пропуски последующих приемов. Другими причинами явились социальное «клеймо» и раздел и продажа еды, согласно результатам обсуждения в фокус-группе.

Заключение: Наблюдаемый уровень приверженности программе «Еда по рецепту» среди респондентов, участвующих в программах диетологического вмешательства, был низким. Основными факторами, связанными с низкой приверженностью, были: низкий уровень образования, недостаточные знания преимуществ RUF, долгая продолжительность программы, потребление более двух предписанных пакетов RUF в день и неосведомленность о продолжительности программы. Следовательно, консультирование пациентов о преимуществах программы, включая планы лечения, скорее всего, повысит уровень приверженности лечению.

Translated from English version into Russian by Elena McDonnell, through

TRANSLATORS
WITHOUT BORDERS

Factores que influyen en la adhesión al programa de Alimentación por Prescripción médica de pacientes adultos con VIH positivo en Addis Abeba, Etiopía: Un estudio sectorial basado en una institución

Mesrach Ayalew Kebede y Dr. Jemal Haidar

Resumen

Antecedentes: Las intervenciones nutricionales han tenido éxito en el tratamiento del VIH y del SIDA y muchos pacientes que participaron en los programas de nutrición han mejorado notablemente su peso corporal y la salud general. Una manera de abordar la malnutrición entre los pacientes con VIH / SIDA es a través del programa de Alimentación por Prescripción. Muchos estudios han explicado los resultados del tratamiento después del aporte de suplementos alimentarios terapéuticos, pero pocos estudios se encuentran disponibles sobre los niveles de adhesión y los factores asociados a este tipo de programas. Los resultados de este estudio serían, por lo tanto, contribuir al conocimiento existente sobre la adhesión a la Alimentación Suplementaria / Terapéutica Lista para Usar (RUF, por su sigla en inglés) en Etiopía.

Objetivo: Evaluar los factores que influyen en la adhesión al programa de Alimentación por Prescripción entre los adultos VIH positivo (VIH+) inscritos en los programas de nutrición en los centros de salud de Addis Abeba.

Métodos: El estudio sectorial se llevó en las instalaciones de salud de Addis Abeba, entre febrero y junio de 2013 y se complementó con métodos cualitativos. Se inscribió un total de 630 pacientes adultos VIH positivo procedentes de 34 centros y se midió su nivel de adhesión a RUF utilizando la Escala Morisky de Adhesión a la Medicación de 8 ítems (MMAS-8). Los datos se ingresaron en Epi Info versión 3.5.1 y se analizaron utilizando SPSS versión 20. La puntuación total en los rangos de 0 a 8 de MMAS-8, con puntuaciones de <6, 6 a <8 y 8, refleja una adhesión baja, media y alta, respectivamente. Los pacientes que tenían una tasa de adhesión baja o moderada fueron considerados no adherentes. Se analizaron los factores asociados con un nivel de adhesión utilizando múltiples regresiones logísticas binarias.

Resultados: Seiscientos pacientes participaron en el estudio cuantitativo con una tasa de respuesta del 95% y el nivel de adhesión que se encontró fue del 36,3%. Con excepción de la situación educativa, otras variables sociodemográficas no tuvieron efecto significativo en la adhesión. Los que conocían los beneficios del programa de Alimentación por Prescripción tenían 1,78 veces (AOR = 1,78, IC 95% = 1,22-2,60)

más probabilidades de adherirse al tratamiento que los grupos referentes. Por otro lado, los pacientes que no estaban tomando RUF durante más de cuatro meses fueron 61,0% (AOR = 0,39, IC 95% = 0,24 a 0,63), 47% (AOR = 0,53, IC 95% = 0,33 a 0,85), y 62,0% (AOR = 0,38, IC 95% = 0,22 a 0,68), respectivamente, menos propensos a adherirse al tratamiento que los demás participantes. Según los resultados cuantitativos, las principales razones de la falta de adhesión fueron no gustarles el sabor de la comida y faltar a las citas de seguimiento. El estigma y la distribución y la venta de alimentos fueron las otras razones, como se deduce de las conclusiones de las pláticas con los grupos focales (FGD).

Conclusiones: El nivel de adhesión al programa de Alimentación por Prescripción observado entre los encuestados que participaron en el programa de intervención fue bajo. Los principales factores identificados con una baja adhesión fueron un nivel de educación bajo, la falta de conocimientos sobre los beneficios de RUF, la duración del programa, el consumo de más de los dos sachets de RUF prescritos por día y no estar informados sobre la duración del tratamiento. Por lo tanto, aconsejar a los pacientes sobre los beneficios del programa, incluidos los planes de tratamiento, probablemente contribuiría a la mejora de la adhesión.

Translated from English version into Spanish by Susana Rosselli, through

TRANSLATORS
WITHOUT BORDERS
